# Analysis of Adrenocorticotropic Hormone and Cortisol Levels in Acute Respiratory Distress Syndrome COVID-19 Patients

**DOI:** 10.1155/2022/3191285

**Published:** 2022-09-30

**Authors:** Ferdy Royland Marpaung, Victoria Mayasari, Hanna Sidjabat, Sidarti Soehita, Bambang Pujo Semedi, Ida Parwati, Titin Andri Wihastuti, Agustin Iskandar, Aryati

**Affiliations:** ^1^Department of Clinical Pathology Dr. Soetomo Hospital/Faculty of Medicine, Universitas Airlangga, Surabaya, Indonesia; ^2^Menzies Health Institute Queensland, Griffith University, Queensland, Australia; ^3^Department of Anesthesiology and Reanimation Dr. Soetomo Hospital/Faculty of Medicine, Universitas Airlangga, Surabaya, Indonesia; ^4^Department of Clinical Pathology, Faculty of Medicine, Universitas Padjajaran, Bandung, Indonesia; ^5^Department of Biomedical Science, Faculty of Medicine, Universitas Brawijaya, Malang, Indonesia; ^6^Department of Clinical Pathology, Faculty of Medicine, Universitas Brawijaya, Malang, Indonesia

## Abstract

**Objective:**

SARS-CoV-2 infection may cause multiple organ failure. However, scarce information can be found on the impact on the endocrine system. This study was conducted to determine plasma Adrenocorticotropic hormone (ACTH) and plasma cortisol levels in a cohort of COVID-19 patients with Acute Respiratory Distress Syndrome (ARDS).

**Methods:**

A prospective cohort study was conducted on COVID-19 patients who manifested ARDS and were admitted to the ICU of Dr. Soetomo Tertiary Hospital, Surabaya, Indonesia. Morning plasma ACTH and plasma total cortisol were measured on 45 recruited patients. The outcome of the patient was justified based on the survivance on days 7th and 30th during the follow-up with groupings of surviving for survived patients and nonsurvive for deceased patients.

**Results:**

The ACTH and cortisol median were 1.06 (0.5–64.57) pg/mL and 17.61 (0.78–75) *μ*g/dL, respectively. Both parameters were assembled to allow the allocation of the 45 subjects into the survive and nonsurvive groups. There was a moderate correlation between ACTH and cortisol levels in all groups (*r* = 0.46, *p* < 0.002) and particularly ACTH and cortisol levels in COVID-19 patients who survived on the 7th-day and 30th-day follow-up (*r* = 0.518 and *r* = 0.568, respectively, with *p* < 0.05). It is important to note that there was no correlation for an individual parameter, either ACTH only or cortisol only, compared to the outcome among patients with various comorbid.

**Conclusion:**

ACTH or cortisol alone has no correlation to the outcome of these patients. Therefore, further study of the potential use of corticosteroid treatments guided by ACTH and cortisol levels in reducing the risk of ARDS warrants further investigation.

## 1. Introduction

SARS-CoV-2 viral infection has the potential to cause multiple organ failures such as the heart (myocarditis), liver damage, and kidney failure [1]. There are still very limited clinical studies about the impact of this disease on the endocrine system. However, viremia supports the evidence of the possible effects of the virus on the endocrine systems [2, 3]. The adrenocorticotropic hormone (ACTH) and cortisol levels which might influence ARDS in COVID-19 patients are unclear. The research mentioned a condition called critical illness-related-corticosteroid insufficiency (CIRCI) in ARDS [4–6]. A recent experiment by Tan et al. verified evidence of higher plasma cortisol levels in COVID-19 patients compared to non-COVID-19 patients; it was associated with a higher mortality rate amongst COVID-19 patients [7]. Unfortunately, this study did not measure the patient's ACTH levels to ensure the potential dual combination of ACTH and cortisol effects in both COVID-19 and non-COVID-19 patients [7]. Here, we aimed to scrutinize plasma ACTH and cortisol levels in the outcome of ARDS COVID-19 patients.

## 2. Material and Methods

This study has been approved by The Human Ethics Committee of Dr. Soetomo Academic Hospital/Faculty of Medicine, Universitas Airlangga, Surabaya, Indonesia, with ethical clearance registration number 0148/KEPK/II/2021. Patients with positive SARS-CoV-2 Nucleic Acid Amplification Test (NAAT) admitted as ARDS COVID-19 patients at the Intensive Care Unit (ICU) of Dr. Soetomo Tertiary Hospital during the period from January to May 2021 were recruited. Patients underwent standard set blood samples for blood biochemistry analysis drawn after obtaining informed consent. Inclusion criteria included NAAT positive, presence of ARDS, and no corticosteroid treatment prior to the blood collection for the ACTH and cortisol plasma testing. We performed blood collection for ACTH and cortisol plasma testing in the morning (7.00-8.00 am). Diagnosis of ARDS was based on WHO clinical COVID-19 management criteria [8]. In addition, plasma ACTH and total cortisol measurements on the first day of admission to the ICU were also performed. Patients with a history of adrenal disorders, receiving corticosteroid and visually icteric, lipemic, and hemolyzed were excluded. The outcome of survival/nonsurvival patient data was obtained by observation until the 7th and 30th days after plasma ACTH and cortisol examinations.

### 2.1. Patient Demography, BMI, ARDS, and Comorbidities

Patient demography information, i.e., gender, age, body weight, and height, was collected through hospital electronic medical records (eMR). The body mass index (BMI) was calculated from body weight and height. The comorbidities, which include diabetes, heart problems, and kidney problems, were collated from the medical record. All COVID-19 patients received the highest-possible care provided by ICU of Dr. Soetomo to survive and recover. The patients' survivance on days 7th and 30th were used to classify the patients into the survive and nonsurvive groups.

### 2.2. Laboratory Testing

#### 2.2.1. SARS-CoV-2 NAAT Testing

All nasopharyngeal/oropharyngeal swabs were extracted for the viral RNA using magnetic microparticle-based protocol and reagents of Abbott mSample Preparation System RNA (Abbott, USA). The PCR to detect SARS-CoV-2 was performed using Abbott RealTime SARS-CoV-2 Amplification Reagent Kit (Abbott, USA) on Abbott RealTime PCR machine (m2000 Real Time) with SARS-CoV-2 PCR condition assay.

#### 2.2.2. ACTH and Cortisol Measurement

Plasma ACTH was examined using chemiluminescence immunoassay (CLIA) on Maglumi 2000 CLIA System (Snibe Diagnostic, Shenzhen, China) following the manufacturer's recommendation. In addition, cortisol level was measured using ADVIA Centaur Immunoassay System (Siemens, Germany) following the manufacturer's recommendation.

#### 2.2.3. Blood Biochemical Test of ARDS COVID-19 Patients

Hematology test, liver function, kidney function, D-dimer, CRP, and procalcitonin tests were performed on the ARDS COVID-19 patients within the first 24 hours of hospital admission.

### 2.3. Statistical Analysis

The data were collected and analyzed using SPSS (version 21). Shapiro*–*Wilk test was used for normality data testing. Data with abnormal distribution was tested using Mann–Whitney U Test. The results of the Spearman correlation test were used to see the correlation between ACTH and cortisol. Fisher's Exact Test was used to see the correlation between cortisol and outcome categories. Kaplan–Meier is used to seeing survival. The results are significant when *p* < 0.05. Statistical correlation strength is divided into: 0 − <0.2 (very weak), 0.2 − <0.4 (weak), 0.4 − <0.6 (moderate), 0.6 − <0.8 (strong), and 0.8 − 1 (very strong).

## 3. Results

### 3.1. Patients Characteristics

**Figure 1:**
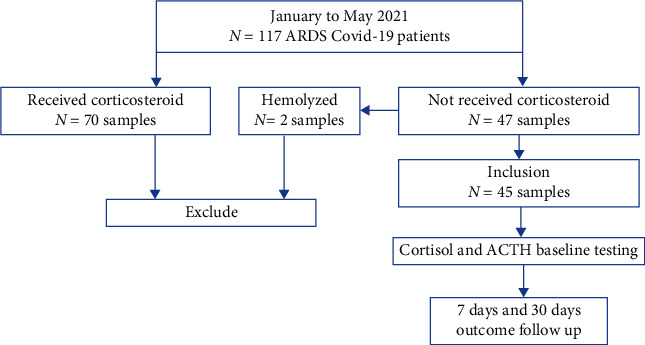
Flow chart ARDS Covid-19 Patients.

We have successfully recruited 117 ARDS COVID-19 patients admitted to the ICU of Dr. Soetomo Hospital for this study. However, of these 117 COVID-19 patients, 70 patients had received corticosteroid treatments prior to the ACTH and cortisol measurements. Therefore, these 70 patients were excluded from the study. In addition, two blood samples of patients within the inclusion criteria were hemolyzed. In total, 45 consecutive patients fulfilled the inclusion criteria for the ACTH and cortisol analysis amongst ARDS COVID-19 infection from January to May 2021 (Figure 1).

Most patients were more than 50 years old (53.86 ± 10.7 years). More than half of the study patients were male (53%). Nearly two-thirds of the patients had one comorbid or more and required a ventilator (66%). Type 2 Diabetes Mellitus (DM) and hypertension were frequently identified among the patients (Table 1). Chest X Rays exhibited bilateral pneumonia in all 45 patients.

Most patients showed leukocytosis (46%), anemia (77%), lymphopenia (62%), neutrophilia (53%), and hypoalbuminemia (Table 2). Data also showed a higher concentration of CRP (95.5%), Procalcitonin (100%), and D-Dimer counts (82%) (Table 2).

### 3.2. ACTH and Cortisol Characteristics

**Figure 2:**
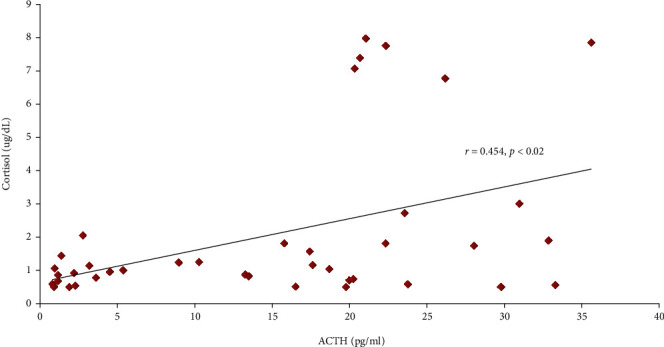
Correlations of ACTH and Cortisol in ARDS COVID-19 Patients.

**Figure 3:**
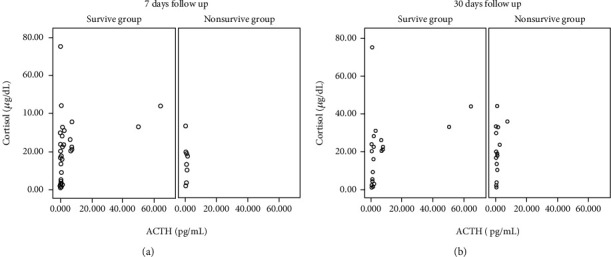
Correlation between ACTH and Cortisol in 7 days (a) and 30 days (b) follow up.

The medians of ACTH and cortisol were 1.06 (0.5–64.57) pg/mL and 17.61 (0.78–75) ug/dL, respectively. There was a moderate correlation between ACTH and cortisol amongst ARDS COVID-19 patients (*r* = 0.46, *p* < 0.002) (Figure 2). In addition, our investigation also revealed a significant correlation between ACTH and cortisol in all patients within the survival group at seven days and 30 days follow-up (*r* = 0.518 and *r* = 0.568, respectively, with *p* < 0.05) but not in the nonsurvive group (Figures 3(a) and 3(b), Table 3). Interestingly, in this study, we found a subject who had very high cortisol levels (>75 ug/dL) and low ACTH (0.5 pg/dL). This subject was a patient with mild ARDS (Pf *ratio* = 352), hyperglycemia (262 mg/dL), high BUN (113 mg/dL) and creatinine (3.25 mg/dL), low creatinine clearance (25 ml/mnt/1.73 m2), high D-Dimer (7,500 ng/mL), hypoalbuminemia (2.6 g/dL), of old age (67 years), and remained alive at 30 days of monitoring. In this consecutive study, we did not exclude the outlier data. The cause of death in nonsurvives cannot be investigated because there was no autopsy data. However, from medical record data, the cause of death in nonsurvives was related to MODS (Multiple Organ Damage Syndrome (MODS) and respiratory failure.

**Figure 4:**
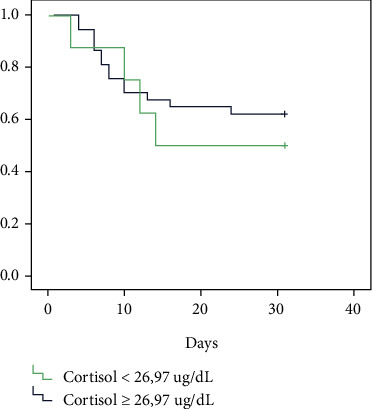
Kaplan–Meier Survival with the cut off of 26.97 ug/dL among COVID-19 ARDS patients.

In correspondence, we used the cut-off 26.92 ug/dL of cortisol plasma and found no difference in survival (Figure 4). We used the same cut-off as the study conducted by Tan et al. which found that a serum cortisol cut of 26.97 *μ*g/dL distinguished high mortality when levels exceeded 26.97 *μ*g/dL. In our observations, with this cut-off, we found no difference in the outcome.

## 4. Discussion

Increased plasma cortisol concentration has been used to indicate physiological stress, often occurring during critical illness and surgery [9]. The mechanism of cortisol metabolism is through activating the hypothalamic-pituitary-adrenal axis (HPA axis), decreasing cortisol metabolism, and reducing cortisol binding globulin (CBG). Enhanced cortisol is a leading mechanism of the body's stress response as it triggers adaptive changes in systemic metabolism, cardiovascular function, and immune regulation [10–12]. In addition, in critically ill patients, cortisol levels may rise overly high, which reflects the severity of the disease. Still, in contrast, the inadequacy of response to stressors will lead to HPA axis failure or adrenal insufficiency [13].

The influence of SARS-CoV-2 in COVID-19 on ACTH and cortisol plasma has not yet been established. Previous research has shown that SARS-CoV has the ability to trigger an immunogenic response to ACTH through mimicry mechanisms [12]. Theoretically, an analogous operation may also take place with SARS-CoV-2, resulting in further morbidity and mortality by inducing cortisol insufficiency [12]. Jefferies et al. investigated ACTH levels in influenza and noninfluenza patients, showing a low concentration of ACTH and normal cortisol levels. The mean ACTH level was 13.5 ± 2.1 pg/mL, with overall plasma ACTH levels in patients being relatively low compared to healthy controls [14]. Similar observation to Jefferies et al. studies, our experiment showed low median morning plasma ACTH levels and normal median cortisol. Wheatland et al. argued that ACTH levels were low owing to the presence of autoantibodies competing with antibodies used in the examination, causing analytical errors in the immunoassay test [12]. Most recently, the study conducted by Tan et al. found that the higher the cortisol level, the higher the mortality rate of patients with COVID-19 compared to non-COVID-19 patients [7]. However, Tan et al. did not include ACTH in the physiological measurement of the HPA axis. Therefore, we initiated this study, which included ACTH and cortisol levels of ARDS COVID-19 patients prior to the corticosteroid treatment.

The most important finding in our study is the correlation between ACTH and cortisol levels in patients with ARDS COVID-19 in the survival group. This finding informs the potential dynamic correlation between ACTH and cortisol levels despite the patients' critical condition.

This study found that ACTH and cortisol had a moderate correlation in subjects' survivance on 7th-day and 30th -day follow-up, but not in subjects who were deceased on 7th and 30th-day follow-up. This discovery supports that an equilibrium state of the HPA axis potentially impacts the outcome of ARDS COVID-19 patients. Furthermore, this study's analysis recorded baseline plasma ACTH and plasma cortisol levels in COVID-19 patients with ARDS with a 30-day outcome.

In contrast, no correlation was found between ACTH or cortisol individual levels on the outcome of COVID-19 patients with ARDS. No correlation was also observed in the patients who were followed up on the 7th and 30th days. Similar results were shown in a study by Leow et al. on 24 post-SARS patients. Leow et al. showed that 83.3% of individuals had central hypocortisolism marked by low levels of ACTH and cortisol. A total of 16.7% of patients had hypocortisolism with plasma ACTH above the upper reference limit of 11 pmol/L (50 pg/mL) [15].

There are several limitations to this study. The setting of this study was intended to see a picture of the relationship between ACTH and cortisol at various levels of ARDS. Therefore, there was no comparison of ARDS patients with non-ARDS. This experiment only performed a baseline sample when ARDS COVID-19 patients were admitted to the ICU. In fact, COVID-19 patients may experience fluctuations in the degree of ARDS from mild to severe or vice versa. These fluctuations were observed during the day-to-day patient management at Dr. Soetomo Hospital. However, investigation of the ACTH and cortisol levels concerning these fluctuations warrants further research.

Further research by having a control group of non-ARDS and non-COVID-19 patients is also recommended to strengthen the findings in this study. This study also did not adjust the various conditions that could affect outcomes. Therefore, we suggest adjusting the analysis of ACTH and cortisol levels in COVID-19 patients with various confounding factors such as hyperglycemia, diabetes mellitus, hypertension, and ongoing treatment for patients' comorbid conditions. Following our findings in this study, we are currently observing the physiological effect of glucocorticoids on ARDS COVID-19 patients by monitoring their ACTH and cortisol levels. We hope the follow-up study will further provide a better understanding of the management of COVID-19 patients for better patient outcomes.

## 5. Conclusions

In conclusion, the most important finding in our study is the correlation between ACTH and cortisol levels in patients with ARDS COVID-19 in the survival group. This finding informs the potential dynamic correlation between ACTH and cortisol levels despite the patients' critical condition. ACTH or cortisol alone has no correlation to the outcome of these patients. Further study of the potential use of corticosteroid treatments guided by ACTH and cortisol levels in reducing the risk of ARDS warrants further investigation.

## Figures and Tables

**Table 1:** Demographic baseline characteristic and clinical outcomes of ARDS COVID-19 Patients.

Parameter	Patients (*n* = 45)
Age (years)	
Mean ± SD	53.86 ± 10.7

Sex	
Male Female	24 (53.3%) 21 (46.7%)

BMI	
Mean ± SD	28.19 ± 6.258

Length of symptoms before COVID-19 confirmed diagnosis (days)	
Median (min-max)	7 (0–21)

Length of symptoms before ARDS (days)	
Median (min-max)	10 (3–22)

Number of comorbid	
1 comorbid 2 comorbid 3 comorbid 4 comorbid	21 (60%) 9 (25.7%) 4 (11.4%) 1 (2.9%)

Comorbid	
Type 2 DM Hypertension Obesity CHD CKD (ESRD) Other	18 (40%) 18 (40%) 10 (24.4%) 2 (4.4%) 2 (4.4%) 4 (8.9%)

Survival	
7 days 30 days	37 (82%) 27 (60%)
Ventilator Nonventilator	30 (66,7%) 15 (33%)

Note: BMI = Body Mass Index; CHD = Cardiac Heart Disease; CKD = Chronic Kidney Disease; ESRD = End State Renal Diseases.

**Table 2:** Laboratory result characteristics of ARDS Covid-19 patients.

Parameter	*N* = 45
SOFA score (mean ± SD) SOFA ≥2	5.09 ± 3.125 43 (95%)

Leukocyte (x10^3^/uL) (mean ± SD) >11 x 10^3^/uL <4 x 10^3^/uL	13.938 ± 5.707 21 (46%) 2 (4%)

Hb (g/dL) (mean ± SD) <12 (g/dL)	11.43 ± 2.349 35 (77%)

Thrombocyte (/uL) (mean ± SD) >450.000/uL <150.000/uL	305.444 ± 136.190 5 (11%) 4 (8%)

Absolute lymphocyte (/uL) (mean ± SD) <500/uL	950 ± 615 28 (62%)

Absolute neutrophil (/uL) (mean ± SD) >8000/uL	13.492 ± 10.541 24 (53%)

Neutrophil to lymphocyte ratio (NLR)	
(mean ± SD) >5.8	19.88 ± 18.46 36 (80%)

D-dimer (ng/mL) (mean ± SD) >1000	3.818 ± 3.806 37 (82%)

C-reactive protein (CRP) (mg/dL)	
(mean ± SD) > 1 mg/dL	12.13 ± 12.0 43 (95.5%)

Procalcitonin (ng/mL)	
Median (min – Max) > 0.1	0.5 (0.1 – 100) 45 (100%)

Early warning system	
Mean ± SD > 10	12.2 ± 1.8 40 (88%)

SGOT (IU/L)	
Mean ± SD Increased (2xULN)	52 ± 33 11 (27%)

SGPT (IU/L)	
Mean ± SD Increase (2xULN)	47.53 ± 35.071 6 (13%)

Albumin (g/dL)	
Mean ± SD < 3.5 g/dL	2.79 ± 0.31 45 (100%)

BUN (mg/dL)	
Mean ± SD > 40 mg/dL	37.39 ± 37.34 15 (33%)

Creatinine (mg/dL)	
Mean ± SD > increase	2.21 ± 2.62 15 (33%)

Creatinine clearance (ml/minute/1.73m^2^)	
Mean ± SD < 15	82.78 ± 54.67 5 (11%)

Random blood glucose (mg/dL)	
Mean ± SD > 200	211.82 ± 85.77 23 (51%)

PF ratio	
Mean ± SD	179.36 ± 129.84

Note: SOFA = sequential organ failure assessment score; CRP = C-Reactive Protein; SGOT = serum glutamic oxaloacetic transaminase; SGPT = serum glutamic-pyruvic transaminase; BUN = Blood Urea Nitrogen; PF = Ratio of arterial oxygen partial pressure (PaO2) in mmHg to fractional oxygen (FiO2).

**Table 3:** Correlation of ACTH and cortisol level.

	*n*	*r*/*r*_*s*_	*p* value
Total	45	0.454	0.002^a^

7 days follow-up			
Nonsurvive	8	-0.028	0.948^b^
Survive	37	0.518	0.001^a^

30 days follow-up			
Nonsurvive	18	0.332	0.178^a^
Survive	27	0.568	0.002^a^

Note: a = Pearson correlation; b = Spearman correlation.

## Data Availability

The statistical data used to support the findings of this study are available from the corresponding author upon request.
